# Bailey on the Eau Medicinale

**Published:** 1811-02

**Authors:** John Bailey

**Affiliations:** Harwich


### *To the Editors of the Medical and Physical Journal.*

### *Bailey on the Eau Medicinale.*

Gentlemen,

From the commencement of your Repository, its pages have been open to the animadversions of candid criticism: hence has arisen its widely diffused and well established fame. A work continued upon principles like this, will not be likely to lose any of its credit.

Although I am no more concerned for the character of the remedy, the effects of which I propose to lay before you, than you are yourselves, yet as the subject has been noticed in your Journal, and as the eyes of the public are turned towards the conduct and opinion of the medical world, it is no more than just, that an *impartial* and *dipassionate* inquiry should take place.

The old maxim, audi alteram partem, holds good in medicine as well as in law.

From much that I had heard and from more that I had read, I considered that the high sounding fame of the new gout remedy would, like many of its precursors, soon "vanish in thin air;" until very lately a small volume being put into my hands, edited by a regular physician of eminence, containing a short history and a list of cases cured by the Eau medicinale. The character of the gentleman who recommended it, and the rank of patients on whom it was tried, banished my scepticism. I determined to make trial of it in the first clear and unmixed case that came in my way. An opportunity soon occurred in the person of a man about fifty years of age, who had had several attacks of regular gout in his feet and hands. Thursday, the 29th ult. was the first day that I saw the patient in his paroxysm; it had come upon him the day before, and was as severe in its attack, and as menacing in its consequences as any one that he before had suffered; it was in his wrist, and from his sensations and former experience promised soon to attack his feet and knees; in this respect he was not deceived, for on the following day he was confined in bed with both his feet wrapped up in flannel, inflamed, swollen, and exquisitely painful; the prospect of passing a dreary winter in pain and distress, lay before him. In this unfortunate condition he begged that I would procure him a bottle of the Eau medicinale; having a good deal of curiosity and more desire to alleviate my patient's distress, I lost no time in complying with his wishes. On Sunday evening, at eight o'clock, he took a tea spoonful, which is about half the bottle, mixed in some water; water; he felt no effect from it until half past two in the morning; at that time a glow of heat broke out and he went into a refreshing sleep, which continued till eight o'clock, when he awoke and expressed himself easy, and slept again till ten; at this time he was well enough to sit up, and whilst drinking coffee he suddenly became sick and vomited; the sickness soon abated: during that and the following day (Tuesday) he was purged smartly, but he had no other ailment; the pain had quitted both his feet and hand. On my visit to him in the evening of Monday, I found him sitting up, cheerful, and free from uneasiness of every description; he said, if it were necessary he could walk out; his feet and hand were a little stiff, but in other respects he was, in his own opinion, as well as ever he was in his life.

This, Gentlemen, is the history of *one* case, and as far as it goes, is certainly in favour of the nostrum. I am not an advocate for quackery, but I feel every disposition to render praise where it is due.

Your correspondent under the pseudonymous title of Reprehensor shews a laudable feeling, in wishing to collate the opinion of medical men, as far as it regards the character and effect of Eau medicinale; there is no doubt, I conceive, that the wishes of this gentleman will be fully satisfied, as the medicine is undergoing a very extensive probation, in the hands of some of the first medical and chemical characters in the metropolis.

I remain, Gentlemen,

Very respectfully, Yours,

JOHN BAILEY.

*Harwich*, *Dec.* 6, 1810.

